# Incidental diagnosis of primary appendiceal signet-ring cell adenocarcinoma after appendectomy for acute appendicitis: a case report

**DOI:** 10.1097/MS9.0000000000001973

**Published:** 2024-03-18

**Authors:** Miao Xie, Fei Li

**Affiliations:** a Department of Gastrointestinal Surgery; b Department of Hepatobiliary and Pancreatic Surgery, Puren Hospital Affiliated to Wuhan University of Science and Technology, Wuhan, People’s Republic of China

**Keywords:** acute appendicitis, appendectomy, appendiceal neoplasms, signet-ring cell adenocarcinoma, treatment

## Abstract

**Introduction:**

Appendiceal signet-ring cell adenocarcinoma (ASCA) is rare and more aggressive in malignant appendiceal neoplasms. The presentation can be appendicitis, which is lack of specific symptom and makes early diagnosis difficult. There is no effective surveillance. Prognosis largely relies on timely detection. We report a case of ASCA incidentally diagnosed through pathological examination after appendectomy for appendicitis.

**Case presentation:**

The patient presented to our department with a progressive right lower quadrant abdominal pain lasting for 3 days. Physical examination revealed rigidity, tenderness, and rebound tenderness on the right lower quadrant. A computed tomography scan showed a thickened, inflamed appendix with peri-appendiceal fat stranding without noticeable appendiceal mass at initial evaluation. The diagnosis was considered acute appendicitis, and an appendectomy was performed. The appendix was inflamed, gangrenous and perforated, and no mass was found during the surgery. Surgical specimen was sent for physiological examination, which incidentally detected signet-ring cell in H&E staining. And immunohistochemistry confirmed the diagnosis of ASCA with small amount of neuroendocrine neoplasms.

**Conclusion:**

Early diagnosis of ASCA can incidentally be made on pathological specimen following appendectomy for appendicitis. A routine pathological examination should be emphasized, and appendectomy may not be the endpoint of the treatment. Hemicolectomy and adjuvant therapy might ensue upon the diagnosis of appendiceal neoplasm. The poor prognosis of ASCA makes a timely diagnosis significant. Basic research is promising to unravel the molecular mechanisms of pathogenesis, finding typical tumor markers for screening and novel effective therapies for advanced cases.

## Introduction

Highlights

- Incidental pathological diagnosis of primary appendiceal signet-ring cell adenocarcinoma (ASCA) following appendectomy for appendicitis.
- Appendicitis is the common presentation for ASCA.
- Clinical diagnosis of ASCA is a real challenge.
- The poor prognosis needs further basic research for an optimal treatment.
- Appendectomy may not be the endpoint for acute appendicitis.

The rarity of appendiceal neoplasm somehow makes an early diagnosis difficult, and the malignant biological behavior of typical pathological classification constantly results in a poor prognosis, where further study is needed to devise an appropriate management^1^. The presentation of this rare neoplasm is often appendicitis in as many as 50% cases^2^. Pathological examination of the surgical specimen following appendectomy for appendicitis seems to be a common way to find the tumor. It is estimated that 0.4–1% of all gastrointestinal malignant neoplasms are appendiceal neoplasms^3^. The rate of pathology specimens finding appendiceal neoplasm in any elective or emergency operation are about 0.7–1.7%^2^. The biological behavior of appendiceal neoplasm depends on the pathological classification.

Adenocarcinoma of the appendix is rare and considered malignant, and even rarer and more aggressive is signet-ring cell adenocarcinoma. We hereby present a case of appendiceal signet-ring cell adenocarcinoma (ASCA) incidentally diagnosed on a pathological specimen after appendectomy for appendicitis and aim to emphasize the importance of a routine pathological examination in it, and streamline a specific algorithm of suggested management based on it.

This case report is in line with the SCARE guidelines 2023^4^.

## Case presentation

The patient was a 64-year-old male who presented to our hospital with the major complaint of abdominal distension for 4 days and right lower quadrant abdominal pain for 3 days. There were no noticeable incentives at the onset of the symptoms. The abdominal pain was dull, intermittent at the beginning, mainly located on the right lower abdomen and started to get worse after admission, accompanied by poor appetite, fatigue, and fever. There was no significant past medical condition, no family history of gastrointestinal cancer, and no prior abdominal surgery.

On physical examination, the first sign showed the patient in distress. And his vital signs were as follows: temperature 38.3, blood pressure 100/63 mmHg, heat rate 65 bpm, and respiratory rate 17 breaths per minute. The bowel sounds were present and diminished. Abdominal palpation revealed tenderness and guarding around the right lower quadrant with rebound tenderness.

The laboratory tests demonstrated an increase of leukocytosis with white blood cell count being 12.68×10^9^/l and neutrophil elevating to 82%, indicating inflammation and abdominal infection. And the red blood cell count and hemoglobin (HGB) were within normal limits. Kidney and liver functions were normal. Electrolytes were imbalanced with lower potassium and sodium. Electrocardiogram (EKG) and chest X-ray found nothing significant.

**Figure 1:**
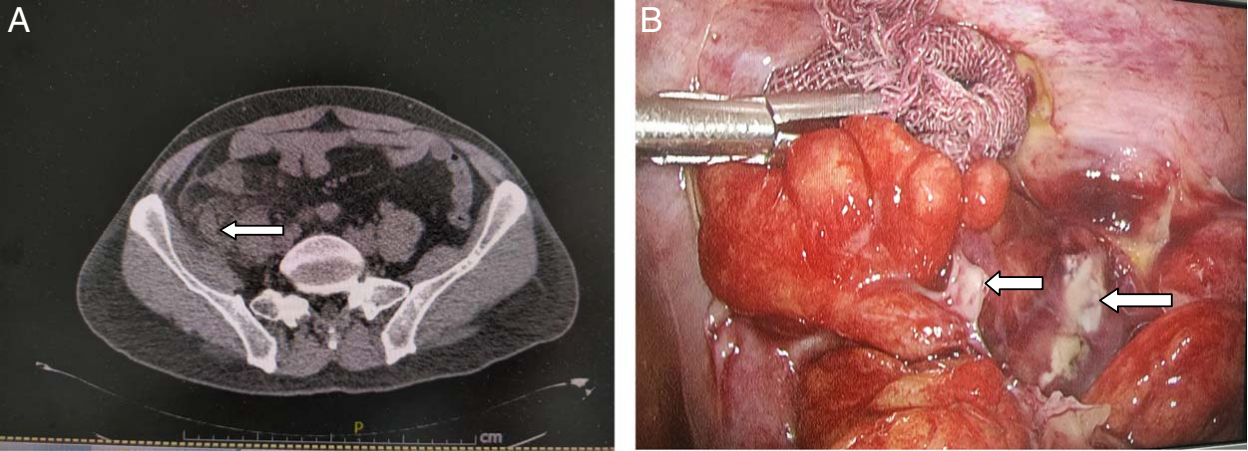
Imaging findings of CT scan and laparoscopy. The appendix was enlarged with peri-appendiceal fat stranding and fluid (arrow ⇦) around it (A); laparoscopy showed purulence (arrow ⇦) around the area of the appendix (B).

After the patient was admitted into our department, we started intravenous fluid resuscitation and antibiotics, and ordered nothing by oral. An abdominal computed tomography (CT) scan was prescribed, and it illustrated a thickened, inflamed appendix and peri-appendiceal fat stranding (Fig. 1A). And there was no abnormal imaging signs on the colon and liver. The patient was simply considered having an acute appendicitis at initial evaluation. A laparoscopy was indicated, and a three-port laparoscopy was then performed in a common fashion. With the camera port inserting around the umbilical and the other two working ports on the left lower quadrant and the suprapubic area, the laparoscopic exploration of the abdominal cavity showed some pus and free fluid around the appendix demonstrating an acute appendicitis with a perforated and gangrenous appendix (Fig. 1B). The size and surface of the liver were normal, and no peritoneum nodule and abnormal mesenteric lymph node were found during the laparoscopic inspection. Then an appendectomy was performed. We carefully suctioned the purulence, and lysed the adhesion of the mesoappendix by harmonic scalpel. The friable appendix was deliberately grasped and elevated by an atraumatic forceps while the mesoappendix with appendiceal artery was divided by harmonic scalpel. The appendix was ligated with 1-0 silk suture at the base and then divided. The specimen was retrieved and then sent for pathological examination. A plastic retrieval bag was used and inserted through the camera port, and the separated appendiceal specimen was carefully grasped and put inside it before pulling the draw-string of the bag through the camera port. At the end, the pelvis was irritated and suctioned before the completion of the surgery.

The patient was continually ordered nil by mouth and administered intravenous fluid resuscitation, nutrition support, and antibiotic on the first day post-surgery. After he started on semi-fluid diet and moved to normal eating, there was no complaint of discomfort. On the fourth day post-surgery, the pathological examination results were received. Unfortunately, appendiceal neoplasm was found.

**Figure 2:**
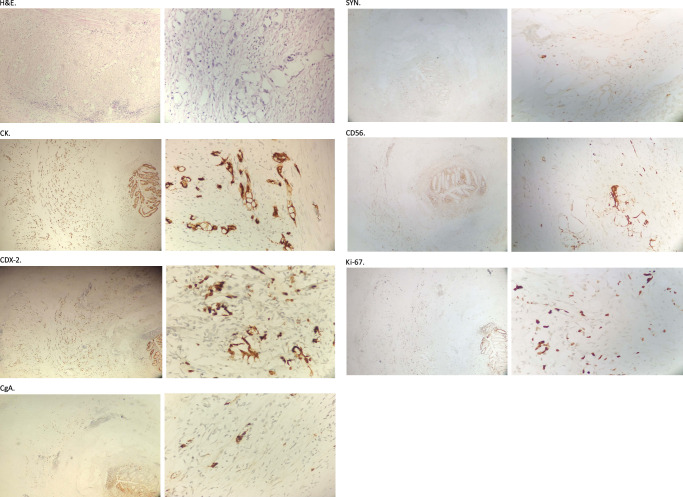
H&E staining and immunohistochemistry results. H&E staining (×10 and ×40 magnification) showed typical signet-ring cell morphology; immunohistochemical analysis demonstrated that CK and CDX-2 (×4 magnification and ×40 magnification) were highly expressed with a high index of Ki-67 and a small amount staining of CgA, SYN, and CD56 (×4 magnification and ×40 magnification).

**Figure 3:**
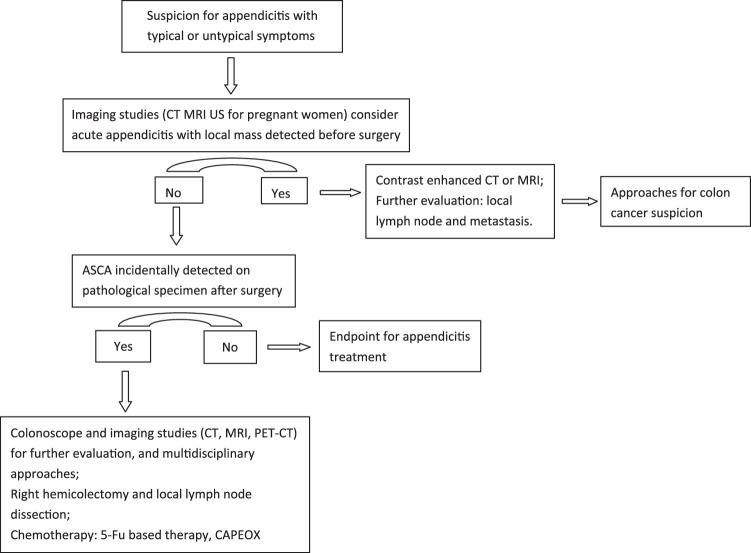
Algorithm for suggested management to incidental diagnosis of ASCA following appendectomy for appendicitis.

H&E staining and immunohistochemistry (IHC) detected mainly signet-ring cell adenocarcinoma with small amount of neuroendocrine neoplasm present in the pathological specimen (Fig. 2). Specifically, cytokeratin (CK) and CDX-2 were positively expressed on the specimen with high index of Ki-67, and CgA and SYN were slightly stained. The current studies show that the prognosis is poor with this typical pathological classification. Thus, we strongly recommended a further management regimen including a colonoscopy, right hemicolectomy, and chemotherapy, but our patient refused it all and required to be discharged after returning to normal diet. He was carefully put on follow-up lists for high recurrence. We streamlined the specific algorithm of managing ASCA not detected at operation based on our case (Fig. 3).

## Discussion

Appendectomy is the standard and least controversial treatment for acute appendicitis, especially perforated appendicitis^2^. Appendiceal neoplasm, though rare, has the odds to be found on pathological specimen after appendectomy for appendicitis^1^. Defining its biological behavior is most important, as always, in planning an appropriate treatment. Pathological classifications of appendiceal neoplasm are diverse and can cause confusion. Broadly, it can be classified into epithelial vs. non-epithelial tumors^1^. Appendiceal adenocarcinoma is a rare epithelial tumor in pathology classification and is considered malignant.

### Signet-ring cell adenocarcinoma: terminology and epidemiology

Signet-ring cell adenocarcinoma, as a rare subtype of adenocarcinoma, is thought to be more aggressive in biological behavior. The 5-year survival was reported to be only 7%^5^. The nomenclature was described as signet-ring cell, simply for the morphological feature that the nucleus was pushed to polarization by the abundant intracellular mucin, and, microscopically, the tumor cell would appear to be like a signet-ring on H&E staining^6^. The demographic data showed that the average age of onset was 60 years for mucinous adenocarcinoma and around 62 years for signet-ring cell adenocarcinoma with a male-to-female ratio of 1:11^7^. Despite the sex discrepancy, our patient’s age was 64, close to the average age. At initial consideration, these data could be useful when preparing for differential diagnosis.

### Carcinogenesis: the molecular basis

The pathogenesis of signet-ring cell adenocarcinoma is not yet defined clearly and needs further research. Like the other well-defined malignant tumors, genetic mutation remains a fundamental principle in it. Pluripotent intestinal crypt epithelial stem cells have the potential function of secreting mucin droplets and forming neuroendocrine secretory granules, and gene mutations in it can contribute to the morphological feature of cytoplasm abundant with mucin vacuoles, which push the nucleus peripherally^8^. The transformation of this type of cell is thought to be linked with the tumorigenesis of signet-ring cell carcinoma. With the advances in sequencing technologies, whole-genome sequencing studies are promising to unravel the complexity of specific gene mutations and underlying mechanisms contributing to the production of the tumors in future studies. Furthermore, it was reported that DNA mismatch repair genetic lesions in Lynch syndrome also stimulate the occurrence of signet-ring cell carcinoma^9^. To elucidate the molecular mechanism that can merit the study of this rare malignant tumor and help find novel therapeutic targets to improve the patients’ outcomes.

### Clinical diagnosis and challenges

The common presentations of appendiceal neoplasm tend to be appendicitis, where the symptoms are variant and non-specific^10^. It may manifest as right lower quadrant pain, vomiting, diarrhea, and weight loss, which are unspecific and cannot directly lead to the diagnosis. Imaging studies, including CT, MRI, and US, are originally used to diagnose acute appendicitis presenting acute abdominal pain. Imaging findings include an inflamed appendix with a thickened wall and peri-appendiceal fat stranding, which also lacks specificity. Appendiceal mass can rarely be observed to raise the suspicion of appendiceal neoplasm before surgery^11^. In our case, the presentation was appendicitis, and this overlapping demonstrations made it extremely difficult to diagnose it before pathological examination of the surgical specimen. Overall, timely diagnosis can be a real challenge since the early-stage symptoms and signs are unspecific, and early surveillance tumor markers for screening are still lacking. The cases are often diagnosed incidentally after appendectomy for appendicitis as shown in our case.

### Pathological diagnosis and tumor staging

Microscopically, the signet-ring cell appearance refers to the pathological classification. To confirm it, IHC serves a diagnostic role^12^. And imaging studies can help distinguish primary vs. secondary lesions, whether the appendiceal neoplasm occurs *de novo*, or is migrated from the other part of the body.

CK is specifically expressed in epithelial cells, and CDX-2, a homeobox gene encoding nuclear transcription factor, has the function of driving gastrointestinal tract adenocarcinoma formation^13,14^. In our case, the IHC positive staining of these two markers together with a high index of Ki-67 and the signet-ring cell H&E staining confirmed the diagnosis of ASCA. There is another comparatively common pathologic type, appendiceal neuroendocrine neoplasm^15^. A detailed discussion is out of the scope of this paper. Of note, the appendiceal neuroendocrine tumor cell was slightly detected with a small amount of staining of the tumor markers of CgA, SYN, and CD56 in the setting of ASCA in our case.

AJCC TNM staging is the most widely used system^16^. Clinical staging is initial evaluation and completed with a CT scan or MRI and positron emission tomography (PET) for pregnant and renal failure patients. After surgery, a pathologic examination adds additional information to the staging (pTNM), which can guide adjuvant treatment. For signet-ring cell, the differentiation level is high grade. In our case, the tumor invasion did not exceed the submucosa without regional lymph node and distant metastasis detected. The pTNM was classified T1N0M0 at its early stage.

### Management and prognosis

Due to its complexity, an appropriate treatment should be individualized and tailored to achieve an optimal effect^17^. The current treatment includes surgical ablation and adjuvant therapy. Multidisciplinary approach is necessary in evaluation and treatment decision. The stage of the tumor invariably dictates the treatment. At an early stage, simple appendectomy can eradicate the lesion from the body with low recurrent rate. Studies suggested that tumors limited in mucosa undergoing a localized resection had a similar 5-year survival with extended surgery^18^. As for an advanced stage, right hemicolonectomy with regional lymph node dissection and chemotherapy might be initiated, as well as cytoreductive (debulking) surgery and hyperthermic therapy, and palliative treatment for spreading and uncuttable tumors. The role of adjuvant chemotherapy remains controversial. The national Comprehensive Cancer Network guidelines recommended 5-fluorouracil-based chemotherapy similar to colon cancer for appendiceal adenocarcinomas^17,19^. Studies suggested capecitabine and oxaliplatin (CAPEOX regimen) especially for signet-ring cell adenocarcinoma. Yet the effects of these treatments remain controversial and more data are needed to investigate the outcome^20^. The role of radiotherapy in ASCA is uncertain. For appendiceal tumor spreading to peritoneum, cytoreductive surgery and hyperthermic intraperitoneal chemotherapy (CRS/HIPEC) are considered the standard treatments^21^. Yet, it remains controversial in peritoneal dissemination from signet-ring cell carcinoma from the appendix^22^.

As far as the current studies go, the prognosis of ASCA is considered poor. In the current phase, a better outcome depends on early detection and prompt treatment. The optimal treatment is still uncertain and awaits for breakthroughs in both clinical and basic researches.

## Conclusions

Appendiceal neoplasm is rare, and the presentations can mimic appendicitis. It can be incidentally found on the surgical specimen of appendectomy for appendicitis. Timely diagnosis is difficult. ASCA is a more aggressive pathological classification with poor prognosis. Upon diagnosis, colonoscopy, right hemicolectomy with regional lymph node dissection, and chemotherapy are suggested. Yet the optimal treatment to improve the prognosis still needs basic and clinical research to devise novel and effective treatment. Appendectomy for appendicitis may not be the endpoint of the treatment.

## Ethical approval

This study is a case report, which is a retrospective review and intended to improve the safety and treatment of similar clinical cases, and we do have our patient’s signed consent to publish this clinical case. Thus, the ethical approval for research and clinical trial should not apply to it.

## Consent

Written informed consent was obtained from the patient for publication of this case report and accompanying images. A copy of the written consent is available for review by the Editor-in-Chief of this journal on request.

## Sources of funding

None.

## Author contribution

X.M. is the first author, responsible for the manuscript writing and data collection; L.F. is the corresponding author and responsible for paper conceptualization and language editing.

## Conflicts of interest disclosure

The author declares no conflict of interest.

## Research registration unique identifying number (UIN)

1. Name of the registry: not applicable.
2. Unique identifying number or registration ID: not applicable.
3. Hyperlink to your specific registration (must be publicly accessible and will be checked): not applicable.

## Guarantor

Puren Hospital and Dr. Li Fei.

## Data availability statement

We declare that all data submitted are available for public access.

## Provenance and peer review

Not commissioned, externally peer-reviewed.
